# Draft Genome Sequences of *Pseudoalteromonas telluritireducens* DSM 16098 and *P. spiralis* DSM 16099 Isolated from the Hydrothermal Vents of the Juan de Fuca Ridge

**DOI:** 10.1128/genomeA.00871-16

**Published:** 2016-08-25

**Authors:** Huan Zhang, Rui Liu, Mengqiang Wang, Hao Wang, Qiang Gao, Zhanhui Hou, Zhi Zhou, Dahai Gao, Lingling Wang

**Affiliations:** a Key Laboratory of Experimental Marine Biology, Institute of Oceanology, Chinese Academy of Sciences, Qingdao, China; b Key Laboratory of Mariculture and Stock Enhancement in North China's Sea, Ministry of Agriculture, Dalian Ocean University, Dalian, China

## Abstract

This report describes the draft genome sequences of two strains, *Pseudoalteromonas telluritireducens* DSM 16098 and *P. spiralis* DSM 16099, which were isolated from hydrothermal vents of the Juan de Fuca Ridge. The reads generated by an Ion Torrent PGM were assembled into contigs with total sizes of 4.4 Mb and 4.1 Mb for DSM 16098 and DSM 16099, respectively.

## GENOME ANNOUNCEMENT

*Pseudoalteromona*s (*Gammaproteobacteria*, *Alteromonadales*, *Alteromonadaceae*) spp. are ubiquitously distributed in the ocean and have become organisms of interest to the fields of ecological and pharmaceutical sciences due to their adaptability to the deep-sea environment and their ability to produce large quantities of biologically active metabolites (1, 2). So far, the draft genome sequences of over 40 *Pseudoalteromonas* strains have been released to public databases, and the complete whole-genome sequences of more than seven strains have been reported (3–5). *P. telluritireducens* DSM 16098 and *P. spiralis* DSM 16099 were isolated from samples obtained in June 1998 on the research vessel *Atlantis* using the deep-ocean-submersible vessel *ALVIN* at the hydrothermal vents located in the Main Endeavor Segment of the Juan de Fuca Ridge in the Pacific Ocean (47.57′N, 129.05′W, depths of 2,000 to 2,200 m) (6). The colonies of DSM 16098 were transparent and creamy, whereas those of DSM 16099 were transparent and colorless. They were selenite- and tellurite-reducing strains, respectively, which could grow in media containing Na_2_SeO_3_ or K_2_TeO_3_ and accumulate metallic selenium or tellurium. In addition, they could also produce capsule- or matrix-like extracellular compounds (6).

The sequencing reads of both strains were generated by the Ion Torrent PGM using a 314 chip version 2 and a 400-bp sequencing kit, which were assembled by the Torrent SPAdes plugin *de novo* assembler version 2.3 and then merged using the CISA contig integrator (7). Protein-coding sequences were predicted by Glimmer software version 3.02 (8), and Ribosomal RNA genes were detected using RNAmmer software version 1.2 (9), while tRNA genes were detected using tRNAscan-SE (10).

The genome of *P. telluritireducens* DSM 16098 consisted of 4,406,320 bases in 65 contigs (*N*_50_ = 165,877 bp and *N*_90_ = 44,541 bp) with a GC content of 40.94%. For *P. spiralis* DSM 16099, the genome consisted of 4,166,507 bases in 87 contigs (*N*_50_ = 108,041 bp and *N*_90_ = 31,480 bp), with a GC content of 40.17%. Both the genome size and GC content were similar to the published data from *Pseudoalteromonas* strains (11). A total of 110 tRNAs and eight rRNAs were predicted for DSM 16098, while 109 tRNAs and 11 rRNAs were predicted for DSM 16099. In DSM 16098, there were 4,278 putative open reading frames (ORFs) with an average size of 892 bp, giving a coding intensity of 84.92%. Meanwhile, 3,990 ORFs were predicted from DSM 16099, with an average size of 910 bp and a coding intensity of 87.22%. A total of 2,869 and 2,750 proteins from DSM 16098 and 16099, respectively, were assigned to cluster of orthologous group families. A comparative genomic study of these two strains is underway to explore the mechanism of selenite and tellurite oxyanion reduction, which could provide new insights into the bioremediation of highly polluted effluents, as well as the biometallurgical applications for their properties as semiconductors.

### Accession number(s).

This whole-genome shotgun project has been deposited in DDBJ/ENA/GenBank under the accession numbers LVCM00000000 for DSM 16098 and LVCN00000000 for DSM 16099. The versions described in this study are the first versions, LVCM010000000 and LVCN010000000.
